# 869. Exploring Attitudes and Beliefs About Burn Care: The Perspective of the Unhoused Patient

**DOI:** 10.1093/jbcr/irag033.346

**Published:** 2026-04-06

**Authors:** Sarah Wang, Dania B Johnson, Jasmine Jin, Haig A Yenikomshian, T Justin Gillenwater, Maxwell B Johnson

**Affiliations:** Keck School of Medicine, University of Southern California, Los Angeles, CA; Keck School of Medicine, University of Southern California, Los Angeles, CA; Keck School of Medicine, University of Southern California, Los Angeles, CA; Division of Plastic and Reconstructive Surgery, Keck School of Medicine, University of Southern California, Los Angeles, CA; Division of Plastic and Reconstructive Surgery, Keck School of Medicine, University of Southern California, Los Angeles, CA; Division of Plastic and Reconstructive Surgery, Keck School of Medicine, University of Southern California, Los Angeles, CA

## Abstract

**Introduction:**

Individuals experiencing homelessness are a uniquely vulnerable population of burn patients. They often sustain more severe burn injuries, have a longer recovery period, and are prone to self-inflicted injuries. Prior research in this population has focused on outcomes, but little is known about their understanding of burn injury and care. This study aims to address this gap, assessing health literacy and exploring challenges faced by unhoused patients in burn recovery.

**Methods:**

This cross-sectional survey study was conducted at a single burn center from 2024-2025. Inclusion criteria encompassed adult (18 or older) patients who were unhoused at the time of admission to the burn unit. Patients who consented to participate were administered a 20-item survey during their inpatient stay consisting of questions regarding living situations, access to care supplies, and knowledge about burn care.

**Results:**

A total of 21 surveys were collected from the inpatient ward. Most respondents were living on the street without any form of shelter (n = 16, 76.2%), while others were residing in shelters (n = 4, 19.0%) or in vehicles (n = 1, 4.8%). A majority had limited prior knowledge of burn wound care and minimal exposure to others with similar injuries. While the most common barriers for patients were access to supplies or knowledge on how to address wounds, almost all were confident in knowing when to seek medical care for a burn wound (n = 16, 76.2%) and expressed trust in the information provided by the healthcare team (n = 20, 95.2%). Regarding educational preferences, most patients favored receiving wound care information through one-on-one explanation (n = 13, 61.9%) and were open to attending wound care classes if offered (n = 15, 71.4%). When asked about burn prevention, over half identified improved shelter or living conditions as the most helpful measure to reduce their risk of future burn injuries.

**Conclusions:**

Unhoused burn patients face significant socioeconomic and educational barriers that complicate recovery. These findings highlight the need for targeted, accessible education on wound care, delivered in a personal and direct manner by healthcare professionals. Building on the strong trust patients express in their care teams, providers can empower individuals not only to manage their own injuries but also to share knowledge within their communities, potentially amplifying the impact of education beyond the individual patient.

**Applicability of Research to Practice:**

Discharge planning for unhoused burn patients should integrate face-to-face education that is practical, resource-conscious, and reinforced with accessible materials. By providing simple instruction and emphasizing prevention strategies, clinicians can improve self-care after discharge. Furthermore, investing in patient education may have a multiplicative effect, as informed patients could help guide peers in recognizing burn injuries, seeking timely care, and utilizing available resources.

**Funding for the study:**

The contents of this abstract were developed under a grant from the National Institute on Disability, Independent Living, and Rehabilitation Research (NIDILRR grant number 90DPBU0007). NIDILRR is a Center within the Administration for Community Living (ACL), Department of health and Human Services (HHS). The contents of this abstract do not necessarily represent the policy of NIDILRR, ACL, or HHS, and you should not assume endorsement by the Federal Government.

